# Assessing the severity of psoriasis through multivariate analysis of optical images from non-lesional skin

**DOI:** 10.1038/s41598-020-65689-4

**Published:** 2020-06-08

**Authors:** Mantas Žurauskas, Ronit Barkalifa, Aneesh Alex, Marina Marjanovic, Darold R. Spillman, Prabuddha Mukherjee, Craig D. Neitzel, Warren Lee, Jeremy Medler, Zane Arp, Matthew Cleveland, Steve Hood, Stephen A. Boppart

**Affiliations:** 1 GSK Center for Optical Molecular Imaging, University of Illinois at Urbana-Champaign, Urbana, IL USA; 2 Beckman Institute for Advanced Science and Technology, University of Illinois at Urbana-Champaign, Urbana, IL USA; 3 GlaxoSmithKline, Collegeville, PA USA; 4 Department of Electrical and Computer Engineering, University of Illinois at Urbana-Champaign, Urbana, IL USA; 5 Department of Bioengineering, University of Illinois at Urbana-Champaign, Urbana, IL USA; 6 Carle Illinois College of Medicine, University of Illinois at Urbana-Champaign, Champaign, IL USA; 7 Department of Dermatology, Carle Foundation Hospital, Champaign, IL USA; 8 GlaxoSmithKline, Stevenage, UK

**Keywords:** Psoriasis, Optics and photonics, Imaging and sensing

## Abstract

Patients with psoriasis represent a heterogeneous population with individualized disease expression. Psoriasis can be monitored through gold standard histopathology of biopsy specimens that are painful and permanently scar. A common associated measure is the use of non-invasive assessment of the Psoriasis Area and Severity Index (PASI) or similarly derived clinical assessment based scores. However, heterogeneous manifestations of the disease lead to specific PASI scores being poorly reproducible and not easily associated with clinical severity, complicating the efforts to monitor the disease. To address this issue, we developed a methodology for non-invasive automated assessment of the severity of psoriasis using optical imaging. Our analysis shows that two-photon fluorescence lifetime imaging permits the identification of biomarkers present in both lesional and non-lesional skin that correlate with psoriasis severity. This ability to measure changes in lesional and healthy-appearing skin provides a new pathway for independent monitoring of both the localized and systemic effects of the disease. Non-invasive optical imaging was conducted on lesions and non-lesional (pseudo-control) skin of 33 subjects diagnosed with psoriasis, lesional skin of 7 subjects diagnosed with eczema, and healthy skin of 18 control subjects. Statistical feature extraction was combined with principal component analysis to analyze pairs of two-photon fluorescence lifetime images of stratum basale and stratum granulosum layers of skin. We found that psoriasis is associated with biochemical and structural changes in non-lesional skin that can be assessed using clinically available two-photon fluorescence lifetime microscopy systems.

## Introduction

Psoriasis, a chronic immune-mediated disease, is characterized by skin and/or joint manifestations along with systemic inflammation^1^, and affects approximately 7.4 million adults in the United States^2^. Many biochemical and immunological markers have been suggested for assessing the severity of psoriasis, but none have as yet been generally accepted^3^. The basic characteristics of psoriasis lesions—redness, thickness, and scaliness provide a means of visually assessing the severity of psoriasis. The current gold standard for assessment of extensive psoriasis has been the Psoriasis Area and Severity Index (PASI)^4^. The PASI is a measure of the average redness, thickness, and scaliness of the lesions weighted by the area of involvement. While the PASI or similarly derived scores are widely used measure in clinics, these do have a number of limitations^5^, one of which is poor sensitivity to changes in relatively small areas of inflammation^6^. PASI and similar scores are difficult to interpret and compare because they entangle the systemic effects of the disease and local severity of individual lesions^7^. Histopathological examination can objectively assess disease severity or the effects of the different treatment methods at the epidermal, dermal, and vascular levels^8^. Histopathology, however, is invasive, time consuming, and can leave permanent scars. Additionally, histopathological examination can only obtain information from a limited number and extent of tissue areas, and does not allow for longitudinal monitoring of disease progression^9^.

In recent years, significant advancements have been made in optical imaging technologies suitable for *in vivo* studies. Among these, multiphoton microscopy (MPM) has been widely used in research studies related to applications in dermatology^9–13^. However, all of the studies employing optical microscopy so far have relied on targeting and imaging of sub-millimeter patches of psoriatic lesions that have inherent structural heterogeneity. In this paper, we present results suggesting that changes associated with psoriasis can be observed in non-lesional skin of psoriasis patients, which is also much less heterogeneous. The aim of this paper is to demonstrate the potential of label-free optical microscopy for the assessment of psoriasis in a clinical setting. In particular, we compared our quantitative optical measurements with clinical scoring systems such as PASI and local inflammation severity (LIS). We utilized a commercial optical medical imaging system (MPTflex CARS, JenLab GmbH, Germany)^14^ based on two-photon excited autofluorescence (AF) and fluorescence lifetime imaging microscopy (FLIM) to obtain optical skin biopsies, non-invasively and label-free, from human subjects diagnosed with psoriasis and eczema, along with healthy volunteers. Furthermore, we collected two separate sets of images from psoriasis patients – one set of images from visibly inflamed sites and the other from a contralateral site on the body that showed no visible signs of inflammation, and appeared otherwise visually indistinguishable from the skin of a healthy volunteer. This paper refers to these two subsets of images as “psoriasis” and “pseudo-control”.

Optical biopsies collected from study subjects presented cellular and sub-cellular structural information based on femtosecond multiphoton (two-photon) excitation of fluorescent biomolecules like nicotinamide adenine dinucleotide (phosphate) (NAD(P)H), flavins, porphyrins, elastin, and melanin. As previously reported, FLIM measures fluorescence lifetimes of fluorophores to generate image contrast, and can be used as a marker for metabolic, biochemical, and morphological changes *in vivo*^10,11,15–17^. Therefore, the FLIM modality was utilized to monitor metabolic changes in the imaged skin regions. Using a fully automated analysis pipeline, statistical features were extracted from the optical biopsies describing structural and biochemical/metabolic properties of skin in each study group. Principal component analysis (PCA) of the extracted features identified clear clusters that were separated along principal component 1 and correlated with skin condition. This suggests that the rotation matrix derived from PCA could be used to form a basis for computing a numerical score for quantifying disease severity. To our knowledge, this is the first clinical study to demonstrate the use of non-invasive optical imaging to monitor and quantify systemic inflammation and biomarkers in psoriasis subjects from images of clear skin areas without visible signs of inflammation. The local effects of psoriasis were observed and evaluated through imaging the psoriasis lesions, while systemic inflammation effects were observed and quantified through imaging and analysis of visibly clear skin areas of psoriasis patients. The study demonstrates the potential of non-invasive optical imaging to obtain “optical biopsy” data through 2-photon fluorescence lifetime imaging. This data can then be further analyzed to assess the changes associated with psoriasis. These unique capabilities can potentially be utilized as a possible tool in clinical settings for monitoring disease progression, or the intended disease regression following drug treatment regimens.

## Results

### Optical biopsy of different skin layers

**Figure 1:**
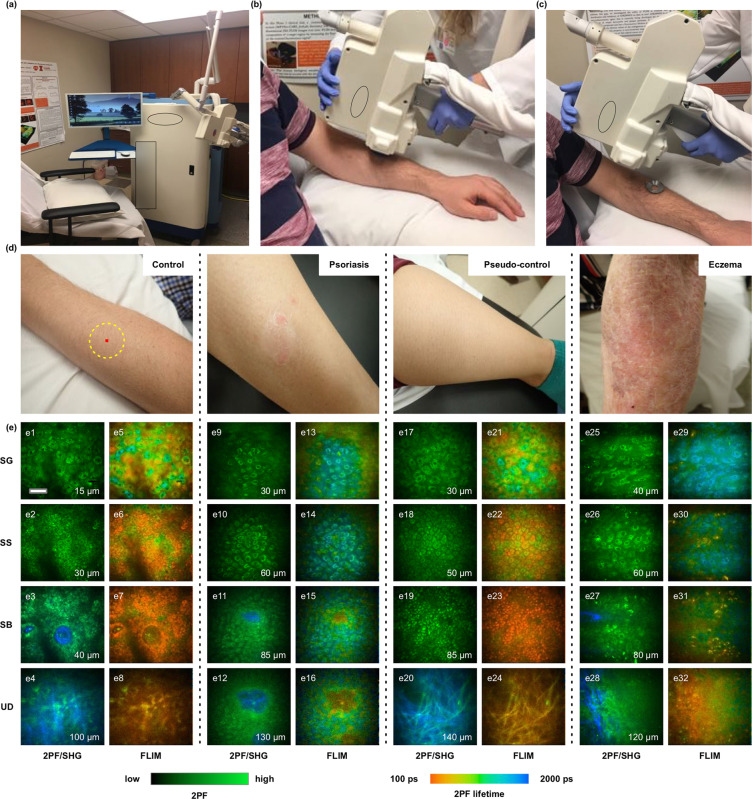
Optical biopsies of different skin layers. (**a**) Commercial optical medical imaging systems utilized for *in vivo* skin imaging. (**b**,**c**) Coupling imaging head to skin. (**d**) Representative digital photos of (left to right) healthy skin of control subjects, lesional skin of subject diagnosed with psoriasis, pseudo-control (non-lesional) skin of subject diagnosed with psoriasis, and lesional skin of subject diagnosed with eczema. (**e**) 2PF/SHG and FLIM images of different skin layers, (SG – stratum granulosum, SS – stratum spinosum, SB – stratum basale, UD – upper dermis). Scale bar in (**e**) represents 40 *μ*m, inset numerical values in (**e**) represent the depth of imaging below the skin surface.

Endogenous fluorophores in human skin such as NAD(P)H, flavins, porphyrins, elastin, keratin, and melanin enabled label-free imaging (Fig. 1a–d) and visualization of cellular and sub-cellular details of different skin layers *in vivo*. As shown in Fig. 1e (2PF/SHG), cellular features in different sub-layers of skin such as the SG, stratum spinosum (SS), SB, and upper dermis (UD) were compared among the 4 study groups. Optical biopsies could detect several skin changes associated with psoriasis. First, keratinocytes in the SG and SS layers of psoriatic lesional skin (Fig. 1e9,e10) appeared inflamed, and perinuclear accumulation of autofluorescence signal was observed in these cells. In addition, cellular features corresponding to various skin layers in psoriatic lesions (Fig. 1e9–e12) were observed in images from deeper depths, compared to control skin (Fig. 1e1–e4) and non-lesional skin (Fig. 1e17–e20), suggesting epidermal thickening in psoriasis. Presence melanin is evident in Fig. 1e3,e7,e19,e23. The intensity images show the melanin caps, and the FLIM images show the short fluorescence lifetime of melanin^18^.

While images presented in Fig. 1e were chosen as representative for each condition, it would be difficult, if not impossible, to accurately determine skin condition through subjective visual analysis of these images. This is a key motivation for developing the automated multivariate analysis presented in this paper.

As shown in previous studies^10,15^, fluorescence lifetime depends on the chemical composition of the imaged region, and is an indicator of the metabolic state of the cells in that region. Figure 1e shows FLIM images of different skin layers from the 4 study groups. Psoriatic skin (Fig. 1e13–e16) shows an increased mean fluorescence lifetime at different skin layers, compared with control (Fig. 1e5–e8) and pseudo-control skin (Fig. 1e21–e24). These observed changes in the FLIM signal suggest biochemical and metabolic alterations occurring in the psoriatic skin. Optical biopsies of skin lesions from eczema subjects (Fig. 1e25–e32) showed optical characteristics similar to the changes observed in psoriatic skin.

### Correlating optical image data with clinical diagnosis

**Figure 2:**
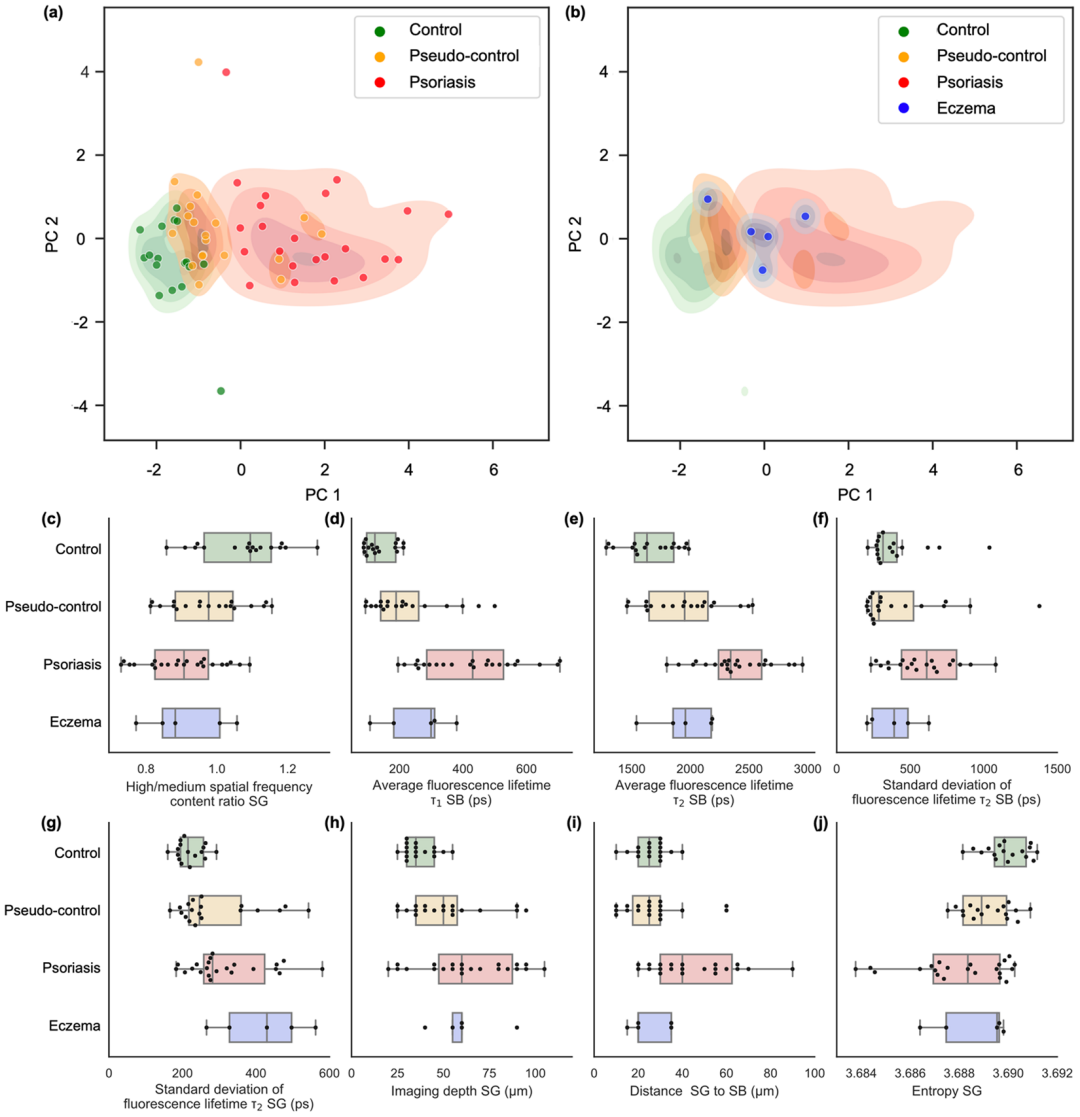
Principal component analysis with kernel density estimates reveals distinct clusters of different skin conditions separated mostly along PC1. (**a**) Low values of PC1 correspond to healthy skin in the control group. Increasingly higher values of PC1 indicate the spectrum of skin conditions transitioning from healthy to pseudo-control to psoriasis. (**b**) Eczema patients form a cluster at the interface between pseudo-control and psoriasis clusters. Outliers can be explained by errors induced by severe artifacts caused by subject movements during image acquisition. Shaded areas show estimated kernel density map for the corresponding groups. (**c**–**j**) Box plots, summarizing the extracted features used for principal component analysis.

The image set was quantified and extracted features were individually scaled to have zero mean and unit variance prior to principal component analysis (PCA). Individually the extracted features can provide some differentiation between control, pseudo-control, psoriasis, and eczema groups (see Fig. 2(c–j)). However, there is still clear overlap within groups if only individual features are considered. This is somewhat expected, as the patient groups are heterogeneous and not controlled for age, gender, or for the precise imaging location on the skin (presence of hair follicles, dermal papillae, etc.).

Figure 2a depicts a scatterplot of the first two principal components that account for 51% of the variance in the dataset. Here, the three groups – control, pseudo-control, and psoriasis subjects, form distinct clusters along a spectrum, which is differentiated predominantly along PC1. When eczema patients are included in the analysis (Fig. 2b), they appear to form a cluster that overlaps with the pseudo-control and psoriasis clusters at their interface.

**Figure 3:**
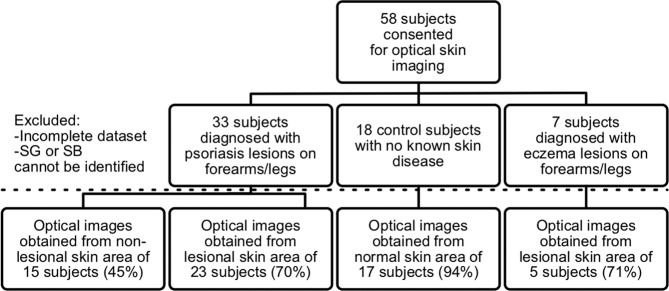
Enrollment, data collection, and usage. Flowchart shows study enrollment, study groups, and the percentage of useful data collected in each study group.

### Correlating optical image data with the clinical scoring system

**Figure 4:**
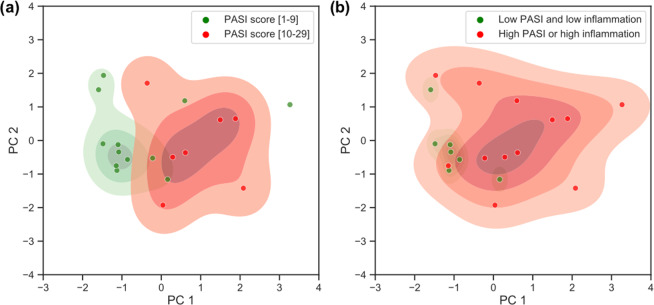
Optical image data correlates with PASI score and LIS score. (**a**) Local severity of psoriasis forms two clusters based on severity. (**b**) Outliers can be explained by high LIS score. Shaded areas show estimated kernel density map for corresponding groups.

We further explored the data extracted from optical images to test how optical data correlated with the PASI scores that were assigned by a dermatologist. For the PCA depicted in Fig. 4a,b, each data point represents a psoriasis patient. Here we used the information from images of inflamed areas and non-inflamed areas (see Methods) to investigate their correlations with PASI scores. Therefore, only patients that had all this information recorded were included in this analysis.

To improve visualization and partially mitigate the ambiguity associated with comparing the slight variations of PASI scores, we assigned patients to one of two groups, each representing about one-half of the cohort: high PASI score [10 to 29] and low PASI score [1 to 9]. As depicted in Fig. 4a, these two groups form two partially overlapping clusters. We further investigated outliers, and found that the local inflammation severity (LIS) also affects the position of each data point, pushing points to the right along the PC1 axis. Figure 4b shows the two newly defined groups that include the LIS assessment along with the PASI score. Better separation of clusters and more granular differentiation between PASI scores is expected if other factors such as age, skin type, and imaging location on the body could be accounted for in larger future studies.

## Discussion

**Figure 5:**
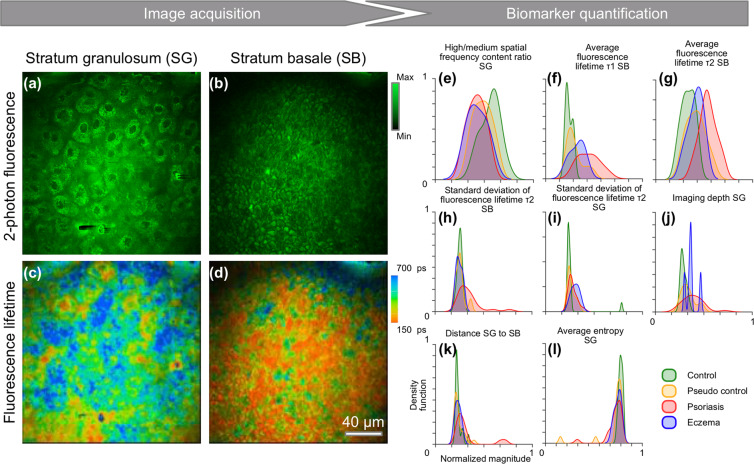
Optical image biomarker quantification. (**a**–**d**) Representative images from a typical data cube that was interrogated during analysis. Each subject was represented by 2-photon fluorescence images from (**a**,**b**) SG and SB layers, and (**c**,**d**) corresponding fluorescence lifetime data, here shown as average fluorescence lifetime. The dimensionality of the dataset was reduced by image feature extraction. (**e**–**l**) Normalized estimated kernel density estimates for the distribution of measured features across the dataset.

The potential of label-free multimodal optical imaging methods in objective and quantitative disease assessment of psoriasis has been explored, following the hypothesis that morphological, metabolic, and biochemical changes detected by optical imaging can be used as quantitative biomarkers for disease severity and progression. From a histological point-of-view, early-to-advanced stages of psoriasis demonstrate epidermal thickening with loss of the granular cell layer, and formations of mounds of parakeratosis, which is thought to result from a markedly shortened cellular turnover time^19^. In line with classical histological analyses, the optical biopsies in this study were able to detect signs of intercellular inflammation and epidermal thickening in psoriatic skin, in comparison to control and non-lesional skin. Additionally, in both psoriasis and eczema, a notably altered 2PF signal distribution was observed within keratinocytes in the SG layer. In skin lesions of both psoriasis and eczema, NAD(P)H autofluorescence, NADH autofluorescence signal accumulated around nuclei, compared with non-lesional skin and control skin, supporting previous demonstrations^20^. NAD(P)H is predominantly located within mitochondria, as previously shown by immunohistochemical studies and fluorescence microscopy^20^. Our results are supported by these previous findings of perinuclear mitochondrial accumulation in inflammatory skin diseases. Thickening of the epidermis in psoriasis has been attributed to the increased proliferation of keratinocytes. These cells proliferate and mature rapidly, with increased mitotic activity, a further indicator of the hyper-proliferative nature of this condition^19^. Analysis of fluorescence lifetime in this study provided a direct insight into the metabolic state of the studied keratinocytes and revealed a shift towards a longer lifetime in all layers of inflamed skin (Fig. 5f,g), indicating an altered metabolic state for the keratinocytes^20^, in addition to other metabolic cell and tissue changes yet to be explored.

In this study, we mostly rely on two parameters to differentiate the contributions to the autofluorescence signal –fluorescence lifetime parameters for two dominant components. Earlier studies demonstrate that multiple endogenous fluorophores such as NAD(P)H, keratin, melanin, etc. can contribute to the autofluorescence signal^18,20–22^. In this study, we were limited to measuring the contributions of two dominant components by the signal-to-noise ratio of measured signals. While measuring two dominant components is clearly beneficial to our study, more advanced instrumentation capable of providing better SNR would permit fitting more components. Alternatively, further analysis using deep learning based approaches could harness correlations between local morphological structures and FLIM data to provide better insight into the biochemical environment.

At present, there are no specific biomarkers that can accurately predict psoriasis progression and therapeutic response. Many efforts have been made to identify psoriasis biomarkers, however, none have yet to be translated into routine clinical practice^23^. In this study, computational analysis of the morphological and metabolic optical image datasets employing a fully automated feature extraction algorithm was used to define a set of statistical features targeted to potential biomarkers of skin changes in the background of psoriasis. These features include changes in mitochondrial distribution, skin scattering, skin thickness, and local metabolism levels detected by FLIM.

We found that the several key features were contributing towards differentiation of different subjects. Features derived from fluorescence lifetime (Average fluorescence lifetime ${\tau }_{2}$ SB, Average fluorescence lifetime ${\tau }_{2}$ SG, Standard deviation of average fluorescence lifetime ${\tau }_{2}$ SB, Standard deviation of average fluorescence lifetime ${\tau }_{2}$ SG) can be associated with the biochemical environment in the areas imaged. Furthermore, skin thickness as measured through the “Distance SG to SB” parameter is a known biomarker for psoriasis. Additionally, Average entropy reflects structural uniformity of the area imaged and skin scattering (as signal-to noise ratio at the imaging site is affected by scattering-induced losses).

As psoriasis results from a complex interaction between genetic, environmental, and immunological factors, each image feature separately could not provide enough separation between different disease groups – control, pseudo-control, and psoriasis groups. However, by combining all the identified optical biomarkers, the three groups could readily be separated, and formed distinct clusters along a spectrum, predominantly along the PC1 axis. To our knowledge, this is the first study to demonstrate the ability to use optical imaging to track systemic inflammation markers that appear in otherwise healthy-looking skin (pseudo-control) in psoriasis patients.

In daily clinical practice, it is often difficult to distinguish certain variants of psoriasis and eczema. This holds especially true for skin lesions on the palm or scalp, and for psoriasis mechanically altered by scratching^24^. With eczema patients included in our analysis, their corresponding optical signatures appear to form a cluster that falls at the interface between the pseudo-control and psoriasis groups, suggesting that both conditions appear as inflamed by optical assessment, with lower levels of inflammation in eczema in comparison to the psoriatic skin. However, no significant separation was observed between eczema and psoriasis subjects due to the limited number of eczema patients. Future studies including larger numbers of eczema subjects are needed for the development of clear differential assessment using these optical imaging techniques.

Combined optical imaging biomarkers (PC1) showed a positive correlation with PASI score (Pearson correlation coefficient = 0.49), the current gold standard clinical scoring system for psoriasis.  A significant correlation was also shown (Pearson correlation coefficient 0.57) when combining the PASI score and the LIS score, supporting the reported limitation of the PASI score alone for its poor sensitivity to changes for relatively small areas of involvement. In studies involving treatment of localized psoriasis plaques, lesion assessments are generally performed that also measure redness, thickness, and scaliness of target plaques^6^. Disease assessment using optical imaging targets specific skin areas objectively, and offers a significant added value to observer-based all body PASI assessment.

In conclusion, we present a fully automated assessment of optical skin biopsies, based on 2-photon fluorescence lifetime microscopy images, from the SG and the SB layers. This optical assessment permits assigning a numerical score that can non-invasively and objectively quantify skin condition and clearly separate control, pseudo-control, and psoriasis groups. This assessment correlates well with the current gold standard clinical scoring system (PASI) and may even be more accurate and sensitive to local disease severity as well. These unique optical imaging capabilities are of immediate relevance to future studies of inflammatory skin conditions such as psoriasis and eczema, and may also be used as a tool for monitoring disease progression or regression in the clinical setting following various drug treatment regimens.

## Methods

### Patient recruitment

A total of 18 healthy human subject volunteers and 40 patients (Fitzpatrick skin type II and III) who were undergoing a routine dermatological exam were enrolled in this study (See Fig. 3). Among the patients, 33 were diagnosed with psoriasis and 7 were diagnosed with eczema. Physicians diagnosed the state of the diseases in each patient and assigned a PASI score and a LIS score for manifestation in the investigated skin areas.

Optical biopsies were acquired from lesional and non-lesional skin areas of psoriasis subjects, lesional skin in eczema subjects, and healthy skin in control subjects. Physician consultation notes were used to collect clinical and demographic information for each subject. The patient imaging protocol was approved by the Institutional Review Board (IRB) at Carle Foundation Hospital (reference number 16090) and the healthy volunteer imaging protocol was approved by the IRB at the University of Illinois at Urbana-Champaign (reference number 11012). Informed consent was obtained from participants prior to any study procedure. All methods were carried out in accordance with the relevant guidelines and regulations.

### Psoriasis Area and Severity Index (PASI) and Local Inflammation Severity (LIS) scores

Psoriasis Area and Severity Index (PASI) scores were assigned by the treating dermatologist by calculating the BSA (Body Surface Area) covered with lesions and making an assessment of the severity of lesions in four body regions: head, upper limbs (right and left), trunk, and lower limbs (right and left). The severity consisted of assessing lesion erythema (redness), induration (thickness), and scaling. The severity score for each region was reached by adding scores for redness, thickness, and scale, each of which was graded from 0 to 4, giving a maximum score of 12. An area and severity score for each region was calculated by multiplying the area score by the severity score (minimum = 0; maximum $6\ast 12=72$). All calculations were combined into a single score (PASI Score). While the PASI score alone is not typically used for clinical decisions, PASI scores of less than 10 indicate mild psoriasis, and scores higher than 10 typically indicate moderate to severe cases^25^.

The Local Inflammation Severity (LIS) Score was assigned to each psoriasis patient by one of the participating dermatologists based on the digital photos of the diseased imaged area, and scored on a scale of 1–10, with 10 being the most severe.

### Optical biopsies

We captured optical biopsy images using the procedure described in^10^. A commercial optical medical imaging system (MPTflex CARS, JenLab GmbH, Germany) was utilized to collect optical biopsies in this study (Fig. 1a). For generating optical images, the excitation wavelength of the femtosecond laser was set to 760 nm and *in situ* laser power was set to 30 mW, which is well below ANSI Z136.1 (2014) safety limits^26^. The light was focused through a 40x 1.35 NA objective. Autofluorescence signals within the spectral range of 405 nm–600 nm were detected. For full details of the imaging system please refer to (Weinigel *et al*.)^14^. MPTflex is a CE marked class 1 M /IIa medical product that has been used in numerous clinical studies in Europe and the U.S.^9,27–29^.

Forty (40) optical sections were taken in 5 *μ*m steps from the stratum corneum (SC) to the upper dermis (UD), down to a depth of 200 *μ*m, with spatial resolutions of <0.5 *μ*m horizontally and <2 *μ*m vertically. The lateral field of view was 200 *μ*m × 200 *μ*m. The time required to acquire each volumetric dataset was approximately 10 minutes per imaging site. We acquired images from a single location per patient in eczema patients and healthy volunteers, and acquired imaged from one location from inflamed and one location from a non-inflamed contralateral site in psoriasis patients. The imaging locations for the psoriasis patients were chosen from an inflamed area without excessive scabbing and another one from a contralateral site. If multiple areas were available, then the area that was most accessible for the optical imaging head was selected. For healthy volunteers, imaging locations on the left dorsal forearm were chosen.

### Image analysis pipeline

After all images were acquired, the fluorescence lifetime information from 2PF images was extracted using SPCImage software (Becker and Hickl GmbH, Germany). A feature extraction algorithm was implemented using Python 3.7 and scientific data libraries Scikit-image, Scipy and Numpy, Pandas, and Scikit-learn were used for data processing and analysis. Matplotlib and Seaborn libraries were used for plotting.

For feature extraction, statistical analysis of whole frames was employed to enable a reproducible data analysis pipeline that did not rely on unreliable segmentation of noisy image data. In particular, the omission of the segmentation step permitted comparison of datasets that were affected by lateral motion artefacts that would otherwise distort the shape of individual cells. Once the depths of interest were selected from the original data cubes, the statistical approach enabled the implementation of an unbiased and fast data analysis pipeline, which did not require manual intervention. After extracting multiple features from the datasets representing each subject (Fig. 5a–d), we evaluated the results and selected the features that were not co-linear, and work well as biomarkers, providing at least some separation between different target groups. Figure 5(e–l) shows the normalized estimated kernel density estimate for the distribution of different features across the entire dataset. Here, we will briefly describe the feature extraction methods.

Fluorescence lifetime information was extracted using SPCImage software with a double-exponential decay model: $f(t)={a}_{1}{e}^{(-t/{\tau }_{1})}+{a}_{2}{e}^{(-t/{\tau }_{2})}$, where *t* is time, *a* is amplitude and $\tau$ is the lifetime of an exponential component. To increase the signal-to-noise ratio, a binning factor of $n=4$ was used, resulting in a moving window of $(2n+1)\cdot (2n+1)$ pixels used for calculating the decay trace at each image pixel. The average lifetimes for two dominant components, ${\tau }_{1}$ and ${\tau }_{2}$, as well as the standard deviation of lifetime distributions within the frame, were extracted and saved for further analysis.

The 2PF images contain information about skin structure with cellular and sub-cellular resolution. Previous studies have suggested that local inflammation causes mitochondrial clustering^30^. In 2PF images, mitochondrial clusters appear as sharp, well-defined structures, increasing the high spatial frequency content of the image. Therefore, we used Fourier analysis to assess a spatial frequency content, measuring a ratio of high-to-medium spatial frequencies for each image. To do this, a two-dimensional fast Fourier transform (2-D Transform (FFT) was calculated for each image, and a radial average of absolute intensity values was computed for pixels (*px*) confined by two annuli representing a medium spatial frequency zone (${r}_{1}=14\,px$, ${r}_{2}=24\,px$) and a high spatial frequency zone (${r}_{1}=24\,px,\,{r}_{2}=36\,px$). Low spatial frequencies were excluded as they do not contain the information about the cellular composition of the skin. Very high spatial frequencies were also excluded as they are severely affected by noise. In this paper, we refer to the average of the ratio between medium and high spatial frequencies as the Fourier parameter.

Entropy is the measure of the degree of randomness in an image. To reduce the impact of uneven illumination profiles across each frame, we first calculated the entropy using a circular structuring element with a radius of 2 *px*, and then computed the average value for the whole frame. Variations in skin thickness were captured by measuring the imaging depth of the SG layer, as well as the distance between the SG and SB layers. The axial depth values were read from metadata that was saved during the imaging sessions. For examining the correlations between optical data and skin condition, we used the following 8 features: Fourier parameter at the SB; average fluorescence lifetime ${\tau }_{1}$ at the SB; average fluorescence lifetime ${\tau }_{2}$ at the SB; standard deviation of fluorescence lifetime ${\tau }_{1}$ at the SB; standard deviation of fluorescence lifetime ${\tau }_{2}$ at the SG; imaging depth at the SG; optical distance from SB to SG, and the entropy at the SG. For examining the correlations between optical data and PASI score, we used the following 5 features: optical distance from the SB to SG in the inflamed area; ratio between optical distances from the SB to SG in the inflamed area and pseudo-control area; ratio of percentages of fluorescence originating from the component with fluorescence lifetime ${\tau }_{2}$ at the SG in the inflamed area and pseudo-control area; ratio of the Fourier parameter at the SB in the inflamed area and pseudo-control area, and the percentage of fluorescence originating from the component with fluorescence lifetime ${\tau }_{1}$ at the SB.
